# Antimicrobial Activity of Selected Medicinal Plants from a Sub-Saharan African Country against Bacterial Pathogens from Post-Operative Wound Infections

**DOI:** 10.3390/medsci9020023

**Published:** 2021-03-31

**Authors:** Enid Owusu, Martin Mensah Ahorlu, Emmanuel Afutu, Amos Akumwena, George Awuku Asare

**Affiliations:** 1 Department of Medical Laboratory Sciences, School of Biomedical and Allied Health Sciences, University of Ghana, Accra 00233, Ghana; martunahorlu@gmail.com (M.M.A.); gaasare@ug.edu.gh (G.A.A.); 2 Department of Medical Microbiology, School of Biomedical and Allied Health Sciences, College of Health Sciences, University of Ghana, Accra 00233, Ghana; emmalineafutu@yahoo.com (E.A.); aakumwena@yahoo.com (A.A.)

**Keywords:** bacteria, herbal, plants, MDR, resistance, susceptibility, wound

## Abstract

Background: Globally, the application of medicinal plants in the management of acute and chronic wounds can be considered a common occurrence in most traditional medicine practices. In view of this, many plants in the tropical and subtropical regions have been screened for their wound-healing activities. Consequently, plants having antimicrobial activity against multidrug-resistant (MD-R) pathogens can be considered great assets. Therefore, this study evaluated ethanolic and aqueous extracts of five medicinal plants (*Psidium guajava*, *Myrianthus arboreus*, *Alchornea cordifolia*, *Momordica charantia*, and *Justicia flava*) for their antimicrobial activities against MD-R bacterial pathogens isolated from post-operative wounds; Methods: This involved the aqueous and ethanolic extraction of the selected medicinal plants. Preliminary phytochemical constituents of the plants were examined. The agar well diffusion method was then used to determine the antibacterial activity of the leaves against reference strains (*Escherichia coli* ATCC 25922, *Salmonella typhi* ATCC 19430, *Pseudomonas aeruginosa* ATCC 27853 and *Staphylococcus aureus* ATCC 25923, and a Methicillin-Resistant *Staphylococcus aureus* strain) as well as the MDR clinical isolates (*E. coli*, *P. aeruginosa*, *S. aureus* and *CoNS*) from the wounds; Results: The preliminary phytochemical analysis of the leaves showed the presence of saponins, phenolics, and reducing sugars in almost all the plants tested. All plant extracts were observed to have some antimicrobial activity against at least one reference strain. For the clinical MDR isolates, *A. cordifolia* from this study showed highest inhibition to growth of all bacteria used. Activity of *J. flava* against *S. aureus* was highest as compared to that of *E.coli* and *P. aeruginosa*. Similar observation was made for *M. arboreus*, *P. guajava* and *M. charantia* where the highest activity was observed against *S. aureus*; Conclusion: This study has mainly shown that *P. guajava*, *M. arboreus*, *A. cordifolia*, *M. charantia*, and *J. flava* exhibits antimicrobial activities against MD-R bacterial pathogens isolated from post-operative wounds. Also, these plants has bioactive phytochemical compounds with potential medicinal values for the treatment of numerous infections. Therefore, these plants may be helpful in the management of acute and chronic wounds, especially in traditional medicine practices.

## 1. Introduction

Since their discovery, antibiotics have been indispensable in the treatment of microbial infections (especially bacterial infections) and have helped in extending the average life expectancy [1]. Globally, antibiotics have had significant useful impact, including the reduction of morbidity and mortality rate caused by bacterial infections in humans, particularly in the developing countries with limited public health infrastructure [2]. Antibiotics have however been overused and misused in the general population, resulting in an increase in antibiotic resistance rates among several microorganisms [1,2].

Hence in recent years, antibiotic resistance has been recognized as a major global health threat [2,3]. Consequently, efforts have been made to counteract the antibiotic resistance menace, while exploring alternative sources of antimicrobial agents, such as medicinal plants [3].

*Psidium guajava* L. (Myrtaceae), *Myrianthus arboreus* P. Beauv. (Cecropiaceae), *Alchornea cordifolia* (Schumach. & Thonn.) Müll.Arg. (Euphorbiaceae), *Momordica charantia* L. (Cucurbitaceae) and *Justicia flava* (Forssk.) Vahl. (Acanthaceae) are among the tropical plants used for the treatment of various ailments [4,5,6,7,8,9,10,11,12,13,14].

*Myrianthus arboreus* P. Beauv. is a dioecious shrub or tree and belongs to the family Cecropiaceae. It is found growing in forest zones of tropical Africa, including Ghana. Extracts of the leaves and leafy shoots of *Myrianthus arboreus* are used in the treatment of dysentery, wounds, diarrhea, boils, dysmenorrhea and incipient hernia [7]. The oil from the leaves is mainly made up of linoleic acids, which maintains the skin’s impermeability to water [7]. This plant has been observed to exhibit activities against some pathogens including *Plasmodia* spp., *Mycobacteria* spp. and *Trypanosoma* spp. [7].

*Alchornea cordifolia* (Schumach. & Thonn.) Müll.Arg. of the family Euphorbiaceae is an evergreen dioecious shrub and grows in some Sub-Saharan African countries including Senegal, Kenya, Tanzania, DR Congo and Ghana [4]. The leaves are normally used as infusions for the treatment and management of some respiratory and intestinal problems whiles the poultice of the leaves is used for the treatment of wounds [4]. The leaves and root bark of *A. cordifolia* are externally applied to treat leprosy and as antidote to snake venom [4]. The leaves, roots and stem bark have been found to contain terpenoids, steroid glycosides, flavonoids, tannins, saponins, carbohydrate, alkaloids, and several guanidine alkaloids [4]. Different extracts of the leaf, stem bark and root of *A. cordifolia* have been shown to have significant antibacterial and antifungal activity. The ethanolic extracts of the leaves and fruits have shown significant antimicrobial activity on a number of pathogens including Trypanosoma spp., helminthes and some bacteria [4].

*Momordica charantia* L. (Cucurbitaceae) is used in the Amazon, parts of Asia and Africa for its numerous benefits in the treatment of skin infections. The fruits and leaves have been observed to contain saponin-like substances and phenolic compounds [5]. The plant has also been shown to contain anti-tumor properties [6]. A leaf tea of this plant has been used for diabetes, to treat menstrual problems, and as anti-viral agent against hepatitis and measles viruses.

*Justicia flava* (Forssk.) Vahl. of the family Acanthaceae is found growing in various locations including disturbed habitat, in full sun or semi shady areas and on a wide range of soil types. It is very common in tropical and southern Africa [14] and used in traditional medicine for the treatment of diarrhea and dysentery, cough, fever, epilepsy, skin infections and disorders, among others [7,8]. According to Agyare et al. [9], in recent times, there seems to be no report on pharmacological activity for *J. flava*, despite its popular use in traditional (herbal) medicine. Regarding its constituents, steroids including campesterol, stigmasterol, sitosterol and sitosterol-D-glucoside have been detected in the leaves and roots of *J. flava* [10,11].

*Psidium guajava* L. (Myrtaceae) is an important medicinal plant in the tropics [13]. The bark is used in the treatment of diarrhea, stomach ache and diabetes whiles extract of the leaves has been described to be used for the treatment of bronchitis, asthma attacks and for the treatment of dysentery [12]. It constituents include tannins from the bark, monoterpenoids and esters from the ripe fruit, essential oil from the leaves, as well as terpenoids from leaves and in the outer portion [14]. Clinical isolates found to show susceptibility towards *P. guajava* include wound isolates of *Proteus* spp., *Staphylococcus* spp. and *Pseudomonas aeruginosa* [14].

These plants are widely available in Ghana and are believed to possess wound healing abilities by the indigenous people of the study location. In addition to these reasons, their documented properties [4,6,7,8,12,13,14] made them suitable candidates for the current study.

Globally, the application of medicinal plants in the management of acute and chronic wounds can be considered a common occurrence in most traditional medicine practices. In view of this, many plants in the tropical and subtropical regions of the world have been screened for their wound-healing abilities [7,14,15]. Despite the growing menace of antibiotic resistance, and the fact that plants are a promising source of antibiotics with possible activity on multidrug-resistant microorganisms [3], the potential of plant extracts as alternative sources of novel antibiotics against wound pathogens have been under-explored, especially in developing countries. Thus, there still exists a number of medicinal plants to be screened in the search for newer, efficacious, and cost effective wound-healing properties.

For example, in Nigeria, Udegbunam and colleagues investigated the wound healing and antibacterial properties of *Pupalia lappacea Juss* [16], *Crinum jagus* [17] and *Stephania dinklagei* [17]. In Ghana, Boakye et al. [18] determined the in vivo wound-healing activity of aqueous aerial part extract of a medicinal plant using the case of only Phyllanthus muellerianus. Also, Agyare and colleagues [14] evaluated the antimicrobial and wound healing potential of two plants; *Justicia flava* and *Lannea welwitschii*.

It is therefore important to explore more plants that are locally widely available and of low cost, as potential sources of novel antibiotics (specifically against wound infected bacterial pathogens) to contribute to effective management of wound infections. To help achieve this goal, the current study evaluated ethanolic and aqueous extracts of five medicinal (*Psidium guajava* (*P. guajava*), *Myrianthus arboreus* (*M. arboreus*), *Alchornea cordifolia* (*A. cordifolia)*, *Momordica charantia* (*M. charantia*) and *Justicia flava* (*J. flava*)) for their antimicrobial activities against multidrug-resistant (MD-R) bacterial pathogens isolated from post-operative wounds.

## 2. Materials and Methods

### 2.1. Study Design, Location and Ethical Considerations

A quantitative experimental research was carried out at the Microbiology Departments, Central Laboratory, Korle Bu Teaching Hospital (KBTH), and the School of Biomedical and Allied Health Sciences (SBAHS) all of the Greater Accra Region of Ghana, as well as the Centre for Plant Medicine Research (CPMR), Mampong of the Ashanti Region of Ghana. In addition to the Microbiology Department of the SBAHS, the Korle-Bu Teaching Hospital, one of the leading national referral center in Ghana and the third largest hospital in Africa, aided in the clinical isolation of the selected bacteria. The CPMR which focuses on research into herbal medicines has an ultramodern extraction laboratory as well as a microbiology laboratory which helped in the preparation and the microbiological analysis of the plant extracts.

The study was conducted in accordance with existing ethical guidelines after receiving ethical clearance from the Ethical and Protocol Review Committee of the College of Health Sciences, University of Ghana with protocol identification number: CHS-Et/M.5-P5.13/2018-2019.

### 2.2. Herbal Preparations

#### 2.2.1. Macroscopic Examination and Preparation of Extracts

The medicinal plants used were *Alchornea cordifolia* (Schumach. & Thonn.) Müll.Arg. (Euphorbiaceae), *Myrianthus arboreus* P. Beauv. (Cecropiaceae), *Psidium guajava* L. (Myrtaceae), *Momordica charantia* L. (Cucurbitaceae) and *Justicia flava* (Forssk.) Vahl. (Acanthaceae). Identification of these plants was ascertained at the Botany Department of the University of Ghana. One kilogram each of the plant leaves was examined macroscopically for any the presence foreign matter (contaminants such as molds, insects and undesirable material) after which they were washed with distilled water and air dried at room temperature (28–32 °C) for 14 days in a shed at the Centre for Plant Medicine Research. The completely dried plant leaves were then pulverized with a pulverizer mill into coarse powder. The herbs used for this study were prepared as herbal extracts and decoctions. Methods used included extraction techniques, maceration and decoction as described by Sukhdev et al. [19].

#### 2.2.2. Ethanolic Extraction

The extraction of the plant material followed the method as performed and documented by Sukhdev et al. [19]. Briefly, two hundred grams (200 g) of pulverized dried leaves (coarse powder) of the plants were weighed out using an analytical weighing scale.

Three and half liters of 70% *v/v* ethanol was added to it and left to macerate for 4 days at room temperature (28–32 °C), with occasional stirring. After the 4 days, the 70% ethanol was decanted and filtered through a Whatman No. 1 filter paper (Sigma-Aldrich Inc., St. Louis, MO, USA). The extracts were then concentrated in an oven with relatively lower heat at 38 °C for 24 h and stored in a refrigerator at 4 °C.

#### 2.2.3. Aqueous Extraction

Two hundred grams each of the dried leaves of the plants (coarse powder) were brought to boil in 3.5 L of distilled water on a hot plate for about one hour thirty minutes and then strained. The aqueous extracts were filtered using Whatman No. 1 filter paper (Sigma-Aldrich Inc., St. Louis, MO, USA), and subsequently cooled for 45 min. After cooling, the extracts were concentrated using the drying oven with relatively lower heat at 38 °C for 24 h and stored in a refrigerator at 4 °C.

#### 2.2.4. Preliminary Phytochemical Investigations

Preliminary phytochemical analysis using standard procedures described by Evans [20] was used to determine the phytochemical constituents present in the plants. Saponins, phenolics, reducing sugar, flavonoids, polyuronides, cardiac glycosides, anthracenosides, triterpenes, phytosterol and alkaloids were determined. Briefly, for reducing sugars, 1 mL of extracts was diluted with water (2 mL) in a test tube. Fehling’s solutions I (1 mL) and Fehling’s solution II (1 mL) were added and heated in a water bath at 90 °C, forming a brick-red precipitate.

For saponins, diluted solution of the extracts (2 mL) was placed in a test tube and shaken for 15 min. A soapy like column of about 2 cm formed above liquid level. For alkaloids, the extracts (15 mL) were evaporated to dryness in an oven at 55 °C and residue dissolved in 10% *v/v* Hydrochloric acid (10 mL). Ten milliliters (10 mL) of 10% *v/v* ammonia solution was added to precipitate the alkaloids and then extracted with ether (15 mL). The ether portion was evaporated to dryness and 1.5 mL of hydrochloric acid added. To 0.5 mL of the acidic solution was added 2–3 drops of Mayer’s reagents forming opalescence precipitate.

To detect cardiac glycosides, anthracenosides and flavonosides, 25 mL of the extracts was mixed in 10% *v/v* hydrochloric acid (15 mL), refluxed for 30 min, cooled and extracted with diethyl ether (36 mL) in portions of 12 mL each. For cardiac glycosides, to a residue obtained by evaporating to dryness extracts (10 mL) was added acetic anhydride (0.50 mL) and chloroform (0.50 mL) and transferred into a dry tube. Concentrated Sulphuric acid (2 mL) was added by means of a pipette at the bottom of the tube forming reddish-brown ring at the contact zone of the two layers. For anthracenosides, the extract (4 mL) was added to concentrated Sulphuric acid (2 mL) and shaken with 25% *v/v* ammonia solution (2 mL) forming cherished-red solution on the top layer. For, flavonosides the residue obtained by evaporating the extracts (5 mL) was heated in 50% methanol (2 mL). Metallic magnesium (0.5 g) and concentrated Hydrochloric acid (5 drops) was added forming a red solution.

### 2.3. Bacteriological Analyses

#### 2.3.1. Identification of Bacteria Isolates

The bacterial isolates were obtained from archived wound swabs from the Microbiology Department of the School of Biomedical and Allied Health Sciences, University of Ghana, Korle-Bu, Accra. Bacterial isolate authentication was done by secondary culture of the archived isolates.

The collected isolates were thawed and cultured on blood and chocolate agar plates. Differential media of cystine–lactose–electrolyte-deficient agar (CLED) (Oxoid Ltd., Basingstoke, UK), deoxycholate citrate agar (DCA) (Neogen Co. Ltd. Lansing, MI, USA), and MacConkey agar (Sigma-Aldrich Inc., St. Louis, MO, USA) aided in distinguishing between Gram negative bacteria that are lactose fermenters (LF) and non-lactose fermenters (NLF). The isolates were ascertained based on colonial morphology, Gram staining and a battery of biochemical reactions, as defined by Ryan and Ray [21].

Characterization of the bacterial isolates was done phenotypically based on colonial morphology, Gram staining and biochemical tests. Below are detailed description of the isolates’ characterization:

*Escherichia coli*: These isolates were characterized based on their presentation as Gram-negative rods in singles, indole-positive, motility-positive and lactose fermentation.
*Pseudomonas aeruginosa*: Characterization was based on their presentation as Gram-negative rods in singles, oxidase-positive and Pyocyanin (green pigment) production.
*Staphylococcus aureus*: These isolates were characterized based on their presentation as Gram-positive cocci in clusters, catalase-positive and coagulase positive.
*Coagulase-negative staphylococci (CoNS)*: Characterization was based on their presentation as Gram-positive Cocci in clusters, catalase-positive and coagulase negative.

#### 2.3.2. Antibiotic Susceptibility Testing of Bacterial Isolates

Identified bacterial colonies were purified and their susceptibility patterns were determined for various antibiotics using a modified form of the Kirby Bauer method [22,23]. Antibiotics tested included ampicillin, tetracycline, trimethroprim/sulfamethoxazole, gentamycin, cefuroxime, cefotaxime, amikacin, ciprofloxacin, levofloxacin, ceftazidime, meropenem, piperacillin/tazobactam, amoxicillin/clavulanic acid, cefepime, colistin, erythromycin, cefoxitin, vancomycin, teicoplanin and clindamycin. These antibiotics were tested because they seem to be common on the Ghanaian market.

The antibiotic susceptibility testing procedure employed is briefly described as follows. The test organism was emulsified in peptone water until the turbidity was comparable with 0.5% McFarland’s standard. A loopful of the suspension was transferred onto a Mueller-Hinton agar plate (Sigma-Aldrich Inc., St. Louis, MO, USA), and then a sterile cotton swab was used to streak the entire surface of the plate. Sterile forceps were used to apply the antibiotic discs to the surface of the agar plate and incubated at 37 °C for 18–24 h. Zone diameters around the antibiotic discs were measured and classified as sensitive or resistant based on the Clinical and Laboratory Standards Institute (CLSI) break point system [24]. Isolates that were resistant to 3 or more antibiotics were labeled as multidrug resistant (MDR) and selected for testing with the plant extracts. Analysis were done in duplicates.

#### 2.3.3. Antimicrobial Activity Evaluation of Plant Extracts by Agar Well Diffusion Method

The agar well diffusion method (CLSI) was used to screen for antibacterial activity of the leaves with reference strains (*Escherichia coli* ATCC 25922, *Salmonella typhi* ATCC 19430, *Pseudomonas aeruginosa* ATCC 27853 and *Staphylococcus aureus* ATCC 25923, and a Methicillin-Resistant *Staphylococcus aureus* strain) as well as the MDR clinical isolates from the wounds (*E. coli*, *P. aeruginosa*, *S. aureus* and *CoNS*). About three to four isolated colonies of similar morphology were picked from the 18–24 h agar plate of pure cultures using a sterile loop, and then inoculated individually into 4 mL peptone broth (Sigma, P0556, Sigma-Aldrich Inc., St. Louis, MO, USA). The density/turbidity of the inoculum was adjusted to 0.5 McFarland turbidity standard, resulting in a suspension of 1.5 × 10^8^ CFU colony forming units.

Mueller Hinton agar plates (Oxoid, CM0337, Oxoid Ltd., Basingstoke, UK) were seeded with the test organisms and the plates left to dry for five minutes. After drying, wells were made in the agar using sterile cork borer measuring 9 mm in diameter. Hundred microliters (100 µL) of the aqueous and ethanolic extract of leaves were dispensed into the labelled wells. The plates were then kept in the refrigerator for one hour for the extract to diffuse into the medium. The plates were then incubated at 37 °C for 24 h (h) and the zones of inhibition were measured in millimeters. Analysis were done in duplicates.

### 2.4. Statistical Analysis

The data collected in the study were entered into MS Excel and analyzed using Minitab software version 15 (Minitab Inc., State College, PA, USA). Descriptive analysis was carried out on the various plant extracts. For the antibiotic susceptibility testing of bacterial isolates, zone diameters around the antibiotic discs were measured and classified as sensitive or resistant based on the CLSI break point system [24]. Isolates that were resistant to 3 or more antibiotics were labeled as multidrug resistant isolates and the proportions of such isolates were also recorded. For the antimicrobial activity evaluation of the plant extracts using agar well diffusion method, the zone diameters around the wells were measured in millimeters and recorded.

## 3. Results

### 3.1. Preliminary Phytochemical Constituents of the Plants

The preliminary phytochemical analysis of the leaves of the five plants showed the presence of saponins, phenolics, and reducing sugars in all the plants with the exception of ethanolic extract of *J. flava* which did not have saponins and the ethanolic extract of *M. charantia* which did not have saponins and phenolics (Table 1). The presence of phytochemical compounds such as flavonoids, polyuronides, cardiac glycosides, anthracenosides, triterpens, phytosterol and alkaloids were not detected in the leaves of most of the plants used in this study in both aqueous and ethanolic extracts (Table 1).

### 3.2. Proportions of Clinical Isolates and Antimicrobial Susceptibility Patterns

A total of 57 bacterial isolates from post-operative wounds were screened. The clinical isolates were in the following proportions; *E. coli* (42.1%, 24/57), *P. aeruginosa* (33.3%, 19/57), *S. aureus* (21%, 12/57) and *CoNS* (3.5%, 2/57). Among these, all the *E. coli* and *CoNS* isolates (24 and 2, respectively) were found to be multidrug resistant (i.e., they were resistant to 3 or more antibiotics) whiles the other isolates displayed varying degree of multidrug resistance (Table 2).

With regards to the antimicrobial susceptibility patterns, for Gram negative clinical isolates tested in this study, *E. coli* isolates showed the highest resistance of 79% (19/24) against cefepime but was highly susceptible to trimethoprim/sulfamethoxazole (100% susceptibility) and meropenem (88% susceptibility). Also *E. coli* isolates recorded resistance of 75% and 71% against amikacin and piperacillin/tazobactam respectively. The resistance recorded for *P. aeruginosa* clinical isolates ranged from 21% (4/19) to 84% (16/19) among the antibiotics tested. The highest resistance of 84% was recorded among three antibiotics, namely; ciprofloxacin, cefepime and colistin (Table 3).

For the Gram positive isolates, *S. aureus*, showed highest susceptibility to standard antibiotics such as ampicillin (92% susceptibility) and vancomycin (75% susceptibility), but highly resistant to amoxicillin/clavulanic acid with 83% (10/12). For the coagulase-negative *Staphylococci* isolates tested in this study, high susceptibility (100%) was observed for all standard antibiotics used, except for clindamycin and cefepime which recorded resistance of 100%, apiece (Table 3). Antibiotic susceptibility pattern of individual isolates is presented in Supplementary File, Tables S1–S4.

### 3.3. Antimicrobial Activity of Aqueous and Ethanolic Extracts against Reference Strains of Bacteria

Regarding the antimicrobial activity of aqueous and ethanolic extracts against reference strains of bacteria, all plant extracts were observed to have some antimicrobial activity against at least one reference strain (Table 4). For *A. cordifolia*, relatively high antimicrobial activity was observed for aqueous extracts with zone of inhibition ranging from 19 to 29 mm and ethanolic extracts with zone of inhibition ranging from 17 to 28 mm. For *J. flava* and *M. arboreus* mean zone of inhibition was zero for both *E. coli* ATCC 25922 and *S. typhi* ATCC 33458 with both aqueous and ethanolic extracts. However, *J. flava* showed a relatively moderate activity against MRSA (zone of inhibition; aqueous = 15 mm, ethanol = 13 mm) and *S. aureus* ATCC 25923 (zone of inhibition; aqueous = 18 mm, ethanol = 16 mm). Similarly, *M. arboreus* showed a relatively moderate activity against MRSA (zone of inhibition; aqueous = 15 mm, ethanol = 12 mm) and *S. aureus* ATCC 25923 (zone of inhibition; aqueous = 0 mm, ethanol = 12 mm).

For *M. charantia*, apart from *E. coli* ATCC 25922 which did not record any activity, zone of inhibition ranged from 10 mm to 18 mm for aqueous extracts and 10 mm to 26 mm for ethanolic extracts against the other isolates (Table 4). Both aqueous and ethanolic extracts of *P. guajava* also did not have activity also against *E. coli* ATCC 25922 but showed activity against the rest of the isolates with zone of inhibition ranging from 7 mm to 21 mm for ethanolic extracts and 8 mm to 26 mm for aqueous extracts. For *P. guajava* no activity was observed with aqueous extracts against *S. typhi* ATCC 33458 (Table 4).

### 3.4. Antimicrobial Activity of Aqueous and Ethanolic Extracts against Clinical Isolates of Bacteria

Table 5 presents results on mean zones of inhibition recorded for the aqueous and ethanolic plant extracts against the clinical bacteria isolates (See Supplementary File Tables S5–S7 for zones of inhibition of individual isolates). For the clinical MDR isolates, *A. cordifolia* from this study showed highest inhibition to growth of all bacteria, with the highest activity observed against *S. aureus* (mean zone of inhibition 21.4 mm = for aqueous extract and 23.4 mm for ethanolic extracts) (Table 5). Activity of *J. flava* against *S. aureus* was highest (6.4 mm for aqueous and 1.6 mm for ethanol) as compared to that of *E. coli* and *P. aeruginosa*. Similar observation was made for the other plants where high activity was observed against *S. aureus*; *M. arboreus* (1.3 mm for aqueous and 0.6 mm for ethanol) and *M. charantia* (9.9 mm for aqueous and 11.1 mm for ethanol). Also, *P. guajava* showed highest activity (16.1 mm for aqueous and 17.6 mm for ethanol) against *S. aureus*, followed by *P. aeruginosa* with zone of inhibition of 2.4 mm for aqueous and 9.2 mm for ethanolic extract (Table 5).

## 4. Discussion

Like many countries in Sub-Saharan Africa, Ghana hosts a number of plants with medicinal properties [25]. However, just a handful of studies have exploited the activity of these plants against wound infected bacterial pathogens [14,18]. For instance, Boakye et al. [18] determined the in vivo wound-healing activity of aqueous aerial part extract of a medicinal plant using the case of only Phyllanthus muellerianus. Agyare et al. [14] also evaluated the antimicrobial and wound healing potential of two plants; *Justicia flava* and *Lannea welwitschii*. However, most of these studies did not include a wide variety of plants and also did not target MDR wound isolates. Also, to the best of our knowledge, there seem to be limited attention given to post-operative wounds in this regard. To help fill this gap, the current study mainly evaluated ethanolic and aqueous extracts of five medicinal plants (*Psidium guajava*, *Myrianthus arboreus*, *Alchornea cordifolia*, *Momordica charantia*, and *Justicia flava*) for their antimicrobial activities against multidrug-resistant (MD-R) bacterial pathogens isolated from post-operative wounds. These low cost and easily accessible plants in Ghana have been suspected by natives to have wound healing properties, however, not all of these plants have been investigated. The current study therefore provides evidence supporting the antimicrobial activity of extracts of these plants against clinical bacterial isolates from wounds; alongside the preliminary phytochemical constituents of these plants.

The preliminary phytochemical screening of the aqueous and ethanolic extracts of the leaves of *Psidium guajava*, *Myrianthus arboreus*, *Alchornea cordifolia*, *Momordica charantia*, and *Justicia flava* in this study showed that the predominant compounds present in the plants were saponins, phenolics and reducing sugars. This observation is similar to several studies with regards to the phytochemical constituents of these plants [14,26,27,28,29]. In terms of health benefit, both saponins and phenolic compounds existing in plants have demonstrated potential medicinal values for the treatment of numerous diseases such as cancers, heart-related illnesses, tumor, bacterial infections, and diabetes [6,30,31]. Therefore, this may account for why these plants have been useful to most people in Ghana (especially those at the countryside) regarding their wound problems; thereby leading to the suspicion of having would healing ability.

Compounds such as flavonoids, anthracenosides and phytosterol were found in some of the plants. Flavonoids are well known for their beneficial effects on health and according to Panche et al. [32], efforts are being made to isolate this ingredient. Flavonoids are considered as an essential component in a variety of pharmaceutical, nutraceutical, medicinal and cosmetic usage [32]. This compound have been found to have anti-oxidative, anti-inflammatory, anti-mutagenic and anti-carcinogenic properties in addition to their ability to modulate key cellular enzyme function [32].

However, other phytochemical compounds, such as alkaloids, triterpenes, and cardiac glycosides were not detected in both aqueous and ethanolic extracts of plants used in this study.

Akbari et al. [31] indicated that the recoveries of bioactive phytochemical compounds from plants are potentially affected by the conditions of extraction methods and different solvent formulations. Other investigators also asserted that the concentration of these compounds, which may or may not be detected in a phytochemical analysis, depends on the nature of the chemical used as solvent in the extraction process, as well the growth and storage conditions [33,34]. Based on all these reasons which may affect the detection of these compounds in the plants, it is therefore not surprising that some of these compounds were not detected. However, the absence of phenolics in *M. charantia* leaves observed in the current study can be considered an interesting finding, since it is against previous knowledge; warranting further investigation. Meanwhile, the current study did not employ HPLC fingerprinting or HPTLC screening for the phytochemical analyses; a limitation that can be considered in future investigations, since these techniques may provide more comprehensive phytochemical investigation.

The antimicrobial investigations from this study showed *E. coli* clinical isolates to have the highest resistance to cefepime, followed by amikacin and piperacillin/tazobactam, but was highly susceptible to trimethoprim/sulfamethoxazole and meropenem. This observation is in contrast to what was recently observed in Ethiopia by Tekele et al. [35] where *E. coli* (from various clinical specimens such as urine, pus, body fluids, sputum, stool, ear and eye discharges) showed the highest resistance to ampicillin followed by amoxicillin with clavulanic acid. Also, although *E. coli* isolates from this study showed the highest resistance to cefepime, followed by amikacin, a study by Yılmaz et al. [36] showed to *E. coli* (from urinary tract infections) as having the highest resistance to ampicillin. In that study [36] cefepime and amikacin recorded the 6th and 10th resistant antibiotics out of the 15 antibiotics they tested. However, similar to this study, Yılmaz et al. [36] observed high susceptibility to meropenem.

*P. aureginosa* from this study was resistant to most antibiotics tested, with highest among them being ciprofloxacin, cefepime and colistin with 84% resistance. Similarly, Yayan et al. [37] have reported an elevated resistance of *P. aeruginosa* and MDR *P. aeruginosa* for cefepime in Germany. In a study by Siddiqua et al. [38] using *P. aeruginosa* from clinical specimens (wound, pus and urine), the isolated pathogens showed resistance to cefepime, ceftriaxone, cefotaxime and gentamicin from 47% to 88%. All the isolates from their study were comparatively better susceptible to meropenem, similar to what is observed in this study with 79% susceptibility. However, ciprofloxacin which showed high resistance in this study also showed better susceptibility in their study. Studies in Ethiopia and India have noted *P. aureginosa* to be highly resistant to many antibiotics [39,40]. Therefore, judicious and rational treatment prescription by physicians to limit further the spread of antimicrobial resistance among the *P. aeruginosa*, as recommended by Siddiqua et al. [38] is highly supported by this study.

*Staphylococcus aureus* isolates showed highest susceptibility to standard antibiotics such as ampicillin (92% susceptibility) and vancomycin (75% susceptibility), but highly resistant to amoxicillin/clavulanic acid with 83% (10/12). This pattern is similar to observations in Ethiopia where *S. aureus* was 100% sensitive to vancomycin [40]. Deyno et al. [41] also observed *S. aureus* to show 75% resistance to ampicillin, however vancomycin recorded 11% resistance compared to 75% resistance recorded in this study. Deyno et al. [41] concluded that *S. aureus* in Ethiopia has gotten notoriously resistant to almost to all of antimicrobial agents in use, including, penicillin, cephalosporins, tetracyclines, chloramphenicol, methicillin, vancomycin and sulphonamides. In the current study, the resistance level observed for amoxicillin/clavulanic acid is bothersome and requires due attention. Meanwhile, studies conducted in Ethiopia and Nepal found *S. aureus* to be susceptible to aminoglycosides, such as amikacin [42,43,44].

For the Coagulase-Negative *Staphylococci* (*CoNS*) isolates tested in this study, high sensitivity rate was observed against almost all antibiotics used; however, for clindamycin and cefepime, there was 100% resistance. After testing *CoNS* strains isolated from a university teaching hospital in China (similar to the site of the current study—Korle-Bu Teaching Hospital) for antibiotic resistance, Ma et al. [45] observed clindamycin to be among antibiotics having resistance rate between 30 and 70%. In that study, antibiotics were placed into three categories based on resistance levels of the *CoNS* strains to these antibiotics: high resistance (resistance rate >70%), including penicillin G, oxacillin and erythromycin; medium resistance (resistance rate between 30 and 70%), including tetracycline, clindamycin, ciprofloxacin, trimethoprim/sulfamethoxazole and chloramphenicol; and low resistance (resistance rate <30%), including rifampicin, ceftizoxime and gentamicin [45]. Also, high resistance rates of *CoNS* have been observed in southern Ethiopia for amoxicillin, amoxicillin-clavunilic acid, ampicillin, and tetracycline as 88.9%, 77.8%, 77.8% and 77.8% respectively [42]. Therefore, the finding of high susceptibility rate against almost all antibiotics used in this study can be considered a positive one since one of the characteristics of *CoNS* is their resistance to multiple antimicrobial agents commonly used for the treatment of *staphylococcal* infections. Also, the high resistance rates of *CoNS* observed in other studies [42,45] and the fact that *CoNS* have become increasingly recognized as important agents of nosocomial infection [45] puts the finding of high susceptibility observed in this study in a promising light.

Generally, possible reasons for these differences in resistance levels observed for all the isolates in this study as compared to other studies can be due to the methods employed for antimicrobial testing, variations in the pattern of indiscriminate usage of antibiotics, patients conditions (isolates from this study were solely from post-operative wound infections) and the nature of bacteria in these countries. Also, some of these drugs (like meropenem) have been on the Ghanaian market for a relatively short period of time compared to others whiles some are also very expensive and usually prescribed for serious infections compared to others. Thus, accounting for the relatively low levels of resistance observed in this study.

Additionally, some of the resistance displayed by the bacterial isolates could be due to biofilm formation. Biofilms provides protection to the bacteria, giving additional resistance power which makes them to not only tolerate harsh conditions but also resistant to antibiotics, which may lead to multi drug resistant, extensively drug resistant and totally drug resistant bacteria [46]. Biofilm-related infections (which included wound infections) are notoriously hard to eradicate and have been a subject of intense scientific research [47]. Consequently, biofilms can have a significant impact on wound healing.

Therefore, biofilms may be considered contributory factors in some of the multi drug resistance observed in this study, although the study did not directly examine the contribution of biofilms.

Another notable limitation of this study is the fact that during the antibiotic susceptibility testing of bacterial isolates, the experiments were done only in duplicates instead of triplicates.

For the antimicrobial activity of aqueous and ethanolic extracts against the clinical isolates, *Alchornea cordifolia* from this study showed highest inhibition to growth of all bacteria used; the highest activity was observed against *S. aureus* for both aqueous and ethanolic extracts whiles *E. coli* was affected more by ethanolic extracts. Similarly, Igbenegu et al. [48] also reported that *A. cordifolia* was active against multi-resistant *S. aureus*. Ebi [49] investigated the antimicrobial properties of methanol extracts of *A. cordifolia* and observed that some fractions of the extracts, notably those containing phenolics and terpenoids, exhibited significant activity against *P. aeruginosa*, *B. subtilis* and *E. coli*. It is worth noting that, in this study, *A. cordifolia* also contained phenolics in both aqueous and ethanolic extracts. However, the isolates used in the current study are MDR and from post-operative wounds unlike that of Ebi [49] which could not be ascertained. Therefore, this plant may be very useful among individuals who may prefer traditional (herbal) medicine in the treatment of their post-operative wounds infected with MDR pathogens, such as those investigated in the current study.

A study by Adeyemi et al. [50] on activity of three different plants (including *A. cordifolia*) on isolates different from those studied in the current investigations showed all their three plant extracts to exhibit varying degrees of activity with *A. cordifolia* being the most active. According to Adeyemi et al. [50] the antibacterial properties of *A. cordifolia* has been extensively studied. Earlier Okeke et al. [51] had shown that it was very active against seventy four bacterial strains studied in vitro. Ajali [52] also made similar observations and the current finding is in line with these earlier reports; however adding that *A. cordifolia* may be useful in management of post-operative wounds. Also, considering the abundance of *A. cordifolia* in most parts of Ghana, this plant would be of benefit to many people in the study location.

Similar observation was made for the other plants where more activity was observed against *S. aureus* for *M. arboreus*, *M. charantia*, *P. guajava* and *J. flava*. According to Agyare et al. [53] extracts of *Myrianthus arboreus* and *Alchornea cordifolia* show some level of antimicrobial activity against common pathogens that cause infections in wounds (especially *S. aureus* and *P. aureginosa*). For *M. charantia*, similarly, Mwambete et al. [54] has shown antimicrobial activity against *Pseudomonas aeruginosa*, *Escherichia coli*, and *Staphylococcus aureus*. These pathogens (*Staphylococcus* sp. and *Pseudomonas* sp.) which are known to be among those commonly associated with wounds infections are also common in Ghana [53]. Therefore, finding various plants to have activity against these pathogens in the current study presents individuals who may prefer traditional medicine in the treatment of their post-operative wounds infected with MDR pathogens with a number of options; thus, readily using which of them is closely available to them.

The antimicrobial activity of the extracts could be attributed to the presence of flavonoids and other secondary metabolites. According to Agyare et al. [53] terpenoids are known to play a role in the antimicrobial activity of some plants due to the possible effect on the nonmevalonate pathway; a pathway that is very essential in most microorganisms (including Gram-negative bacteria) for the synthesis of cell membrane components and as a secondary source of carbon [53]. Therefore, as indicated earlier, future investigations using HPLC fingerprinting or HPTLC screening for all major phytochemicals and finding correlations between such secondary metabolites and the antimicrobial activity is in the right direction.

For *J. flava* Agyare et al. [14] demonstrated that methanol leaf extract was active against *Escherichia coli* ATCC 25922, *Pseudomonas aeruginosa* ATCC 4853 and *Staphylococcus aureus* ATCC 25923. However there seem to be limited studies showing its efficacy against clinical strains, especially those of MDR isolates from post-operative wound infection. Generally, there seems to be just few studies on the medicinal properties of *J. flava* although commonly available in Ghana [14].

In this study, *P. guajava* showing activity against *P. aeruginosa* confirms the observation by Abdelrahim et al. [55], who indicated that, in their study, all clinical isolates tested including that of *P. aeruginosa* were sensitive to extracts of *P. guajava* [55]. This observation confirms the traditional uses of this plant [56], which is also available in most parts of the study location (Ghana). According to Lins et al. [56], *P. guajava* has numerous properties including antioxidant, anti-allergic, anti-inflammatory and antimicrobial. This is likely the reason for which this plant and others investigated in the current study are widely used in traditional medicine for wound healing [4].

## 5. Conclusions

This study has mainly provided evidence to show that *Psidium guajava*, *Myrianthus arboreus*, *Alchornea cordifolia*, *Momordica charantia*, and *Justicia flava* exhibits antimicrobial activities against multidrug-resistant (MD-R) bacterial pathogens isolated from post-operative wounds. It has also showed that these plants have bioactive phytochemical compounds with potential medicinal values for the treatment of numerous infections.

Given the findings of this study, it is recommended that other plants which are being used by people in the treatment of wound infection be scientifically screened against MDR isolates. Also, since topical application of antimicrobials is an efficient therapeutic method of destroying microbial population because of the availability of the drug at the wound sites, further studies aimed at development of the plants investigated in the current study into topical forms for use in management of post-operative wounds is recommended.

## Figures and Tables

**Table 1:** Phytochemical constituents of the aqueous and ethanolic extracts.

Phytochemicals/ Compounds	*P. guajava*		*M. charantia*		*A. cordifolia*		*M. arboreus*		*J. flava*	
	Aq.	Eth.	Aq.	Eth.	Aq.	Eth.	Aq.	Eth.	Aq.	Eth.
Saponins	+	+	+	-	+	+	+	-	+	-
Phenolics	+	+	+	-	+	+	+	+	+	+
Reducing sugar	+	+	+	+	+	+	+	+	+	+
Flavonoids	-	+	-	-	+	-	-	-	-	-
Polyuronides	+	-	-	-	-	-	-	-	-	-
Cardiac Glycosides	-	-	-	-	-	-	-	-	-	-
Anthracenosides	-	+	-	-	+	-	-	-	-	-
Triterpenes	-	-	-	-	-	-	-	-	-	-
Phytosterol	+	+	-	-	+	-	-	+	-	+
Alkaloids	-	-	-	-	-	-	-	-	-	-

*P. guajava*: *Psidium guajava*, *M. charantia*: *Momordica charantia*, *A. cordifolia*: *Alchornea cordifolia*, *M. arboreus*: *Myrianthus arboreus*, *J. flava*: *Justicia flava*; Aq. represents aqueous extracts, Eth. represents ethanolic extracts; + indicates detection of phytochemical agents; - indicate non-detection or absence of phytochemical agents.

**Table 2:** Proportions of multiple drug resistance among the different clinical isolates.

Isolate	Total Number	Number of Multiple Drug Resistant Isolates (MDR) (%)	
		MDR (%)	Non-MDR (%)
*E. coli*	24	24 (100)	0 (0)
*P. aeruginosa*	19	12 (63)	7 (37)
*S. aureus*	12	11 (92)	1 (8)
*CoNS*	2	2 (100)	0 (0)

*CoNS* = Coagulase Negative Staphylococci.

**Table 3:** Antimicrobial susceptibility pattern of Gram-negative and Gram-positive clinical isolates.

Bacterial Isolates			Antimicrobial Agents/Number of Isolates (%)														
Gram Negative	N	S, R	AMP	TET	SXT	GEN	CXM	CTX	AMK	CIP	LEV	CAZ	MEM	TZP	AMC	FEP	CL
*E. coli*	24	S	22 (92)	23 (96)	24 (100)	15 63)	21 88)	22 (92)	6 (25)	21 (88)	22 (92)	20 (83)	21 (88)	7 (29)	19 (79)	5 (21)	-
		R	2 (8)	1 (4)	0 (0)	9 (37)	3 (12)	2 (8)	18 (75)	3 (12)	2 (8)	4 (17)	3 (12)	17 (71)	5 (21)	19 (79)	-
*P. aeruginosa*	19	S	-	-	-	7 (37)	-	-	5 (26)	13 (16)	12 (63)	4 (21)	15 (79)	5 (26)	-	3 (16)	3 (16)
		R	-	-	-	12 (63)	-	-	14 (74)	16 (84)	7 (37)	15 (79)	4 (21)	14 (74)	-	16 (84)	16 (84)
Gram positive			AMP	TET	SXT	GEN	CXM	LEV	TZP	AMC	FEP	ERY	VA	FOX	CC	TEC	CL
*S. aureus*	12	S R	11 (92)	10 (83)	6 (50)	4 (33)	4 (33)	7 (58)	4 (33)	2 (17)	3 (25)	-	9 (75)	-	4 (33)	5 (42)	-
			1 (8)	2 (17)	6 (50)	8 (67)	8 (67)	5 (42)	8 (67)	10 (83)	9 (75)	-	3 (25)	-	8 (67)	7 (58)	-
*CoNS*	2	S R	2 (100)	2 (100)	2 (100)	2 (100)	2 (100)	2 (100)	2 (100)	2 (100)	0 (0)	2 (100)	2 (100)	1 (50)	0 (0)	2 (100)	2 (100)
			0 (0)	0 (0)	0 (0)	0 (0)	0 (0)	0 (0)	0 (0)	0 (0)	2 (100)	0 (0)	0 (0)	1 (50)	2 (100)	0 (0)	0 (0)

*CoNS* = coagulase-negative *Staphylococci*; S = Sensitive; R = Resistant; - = Antibiotic not tested; AMP = Ampicillin; Tet = Tetracycline; SXT = Trimethroprim/sulfamethoxazole; Gen = Gentamycin; CXM = Cefuroxime; CTX = Cefotaxime; AMK = Amikacin; CIP = Ciprofloxacin; LEV = Levofloxacin; CAZ = Ceftazidime; MEM = Meropenem; TZP = Piperacillin/Tazobactam; AMC = Amoxicillin/Clavulanic acid; FEP = Cefepime; CL = Colistin; ERY = Erythromycin, FOX = Cefoxitin, VA = Lancomycin, TEC = Teicoplanin and CC = Clindamycin.

**Table 4:** Antimicrobial activity of aqueous and ethanol plant extracts against the reference strains.

Bacterial Control Strains	*A. cordifolia*		*J. flava*		*M. arboreus*		*M. charantia*		*P. guajava*	
	ZoI (mm)		ZoI (mm)		ZoI (mm)		ZoI (mm)		ZoI (mm)	
	Aqueous	Ethanol	Aqueous	Ethanol	Aqueous	Ethanol	Aqueous	Ethanol	Aqueous	Ethanol
*P. aeruginosa* ATCC 27853	19	17	0	0	0	0	15	10	8	20
*E. coli* ATCC 25922	29	28	0	0	0	0	0	0	0	0
*S. typhi* ATCC 33458	23	20	0	0	0	0	10	12	0	7
MRSA	21	24	15	13	15	12	18	26	22	21
*S. aureus* ATCC 25923	28	27	18	16	0	12	18	13	26	21

ZoI (mm) represents mean zones of inhibition measured in millimeters (mm); *P. guajava*: *Psidium guajava*, *M. charantia*: *Momordica charantia*, *A. cordifolia*: *Alchornea cordifolia*, *M. arboreus*: *Myrianthus arboreus*, *J. flava*: *Justicia flava*; MRSA: Methicillin-resistant *Staphylococcus aureus*.

**Table 5:** Antimicrobial activity of aqueous and ethanol plant extracts against the clinical isolates.

Isolate	N		*A. cordifolia*		*J. flava*		*M. arboreus*		*M. charantia*		*P. guajava*	
			Aqueous	Ethanol	Aqueous	Ethanol	Aqueous	Ethanol	Aqueous	Ethanol	Aqueous	Ethanol
*E. coli*	25	Mean ZoI (mm)	9.2	23.0	0.7	0.0	0.0	0.4	0.3	1.0	0.0	0.3
		STD	7.1	3.1	2.6	0.0	0.0	2.2	1.6	2.9	0.0	1.6
		SEM	1.4	0.6	0.5	0.0	0.0	0.4	0.3	0.6	0.0	0.3
*P. aeruginosa*	20	Mean ZoI (mm)	14.2	16.1	0.4	0.0	0.6	0.0	4.14	4.4	2.4	9.2
		STD	5.3	4.8	1.6	0.0	1.9	0.0	6.0	5.9	4.5	6.9
		SEM	1.2	1.1	0.4	0.0	0.4	0.0	1.3	1.3	1.0	1.5
*S. aureus*	16	Mean ZoI (mm)	21.4	23.4	6.4	1.6	1.3	0.6	9.9	11.1	16.1	17.6
		STD	7.0	1.6	7.3	4.4	3.4	2.3	4.6	4.6	3.4	1.8
		SEM	1.7	0.4	1.8	1.0	0.8	0.6	1.1	1.1	0.9	0.4

N represents total number, STD = standard deviation; SEM = standard error of mean; ZoI (mm) represents mean zones of inhibition measured in millimeters (mm); *P. guajava*: *Psidium guajava*, *M. charantia*: *Momordica charantia*, *A. cordifolia*: *Alchornea cordifolia*, *M. arboreus*: *Myrianthus arboreus*, *J. flava*: *Justicia flava*.

## Data Availability

The data presented in this study are available on request from the corresponding author; enidowusu115@yahoo.com; Tel.: +233-0508-917-569.

## Supplementary Materials

The following are available online at https://www.mdpi.com/article/10.3390/medsci9020023/s1, Table S1: Antibiotic Susceptibility pattern for *Pseudomonas aeroginosa* isolates, Table S2: Antibiotic Susceptibility pattern for *Escherichia coli* isolates, Table S3: Antibiotic Susceptibility pattern for *Staphylococcus aureus* isolates, Table S4: Antibiotic Susceptibility pattern for Coagulase-negative *Staphylococci* isolates, Table S5: Zones of inhibition (mm) recorded for aqueous and ethanol plant extracts against *Pseudomonas aeruginosa*, Table S6: Zones of inhibition (mm) recorded for aqueous and ethanol plant extracts against *Escherichia coli*, Table S7: Zones of inhibition (mm) recorded for aqueous and ethanol plant extracts against *Staphylococcus aureus*.

## Sample Availability

Samples of plant leaves (*Psidium guajava*, *Myrianthus arboreus*, *Alchornea cordifolia*, *Momordica charantia*, and *Justicia flava*) are available from the authors.
